# *Plasmodium falciparum* infection coinciding with the malaria vaccine candidate BK-SE36 administration interferes with the immune responses in Burkinabe children

**DOI:** 10.3389/fimmu.2023.1119820

**Published:** 2023-03-10

**Authors:** Alfred B. Tiono, Nirianne Marie Q. Palacpac, Edith Christiane Bougouma, Issa Nebie, Alphonse Ouédraogo, Sophie Houard, Nobuko Arisue, Flavia D’Alessio, Toshihiro Horii, Sodiomon B. Sirima

**Affiliations:** ^1^ Groupe de Recherche Action en Santé, Ouagadougou (GRAS), Ouagadougou, Burkina Faso; ^2^ Centre National de Recherche et de Formation sur le Paludisme (CNRFP), Ouagadougou, Burkina Faso; ^3^ Department of Malaria Vaccine Development, Research Institute for Microbial Diseases, Osaka University, Suita, Osaka, Japan; ^4^ European Vaccine Initiative (EVI), Universitäts Klinikum Heidelberg, Heidelberg, Germany; ^5^ Department of Molecular Protozoology, Research Institute for Microbial Diseases, Osaka University, Suita, Osaka, Japan

**Keywords:** SE36, malaria blood-stage vaccine, serine repeat antigen, SERA5, *Plasmodium falciparum*, immunogenicity

## Abstract

**Background:**

A vaccine targeting the erythrocyte stages of *Plasmodium falciparum* could play a role in preventing clinical disease. BK-SE36 is a promising malaria vaccine candidate that has shown a good safety profile and immunological responses during field evaluations. It was observed that repeated natural infections could result in immune tolerance against SE36 molecule.

**Methods:**

The primary trial was conducted to assess the safety and immunogenicity of the BK-SE36 in two cohorts of children aged 25-60 months (Cohort 1) and 12-24 months (Cohort 2). Immunization was at full dose (1.0 mL) administered at 0, 1, and 6 months. Blood samples were collected before each vaccination for immunological assessments and detection of *Plasmodium falciparum* infection by microscopy. Blood samples were further collected one month post each vaccination to evaluate immunogenicity.

**Results:**

Of seventy-two (72) subjects that have received BK-SE36 vaccination, 71 had available blood smears during vaccination days. One month post Dose 2, the geometric mean of SE36 antibodies was 263.2 (95% CI: 178.9-387.1) in uninfected individuals compared to 77.1 (95% CI: 47.3-125.7) in infected participants. The same trend was observed one-month post booster dose. Participants uninfected at the time of booster vaccination had significantly higher GMTs compared to those who were infected (424.1 (95% CI: 301.9-595.8) *vs*. 92.8 (95% CI: 34.9-246.6), *p* = 0.002. There was a 14.3 (95% CI: 9.7-21.1) and 2.4 (95% CI: 1.3-4.4) fold-change, respectively, in uninfected and infected participants between one-month post Dose 2 and booster. The difference was statistically significant (*p* < 0.001).

**Conclusion:**

Concomitant infection by *P. falciparum* during BK-SE36 vaccine candidate administration is associated with reduced humoral responses. However, it is to be noted that the BK-SE36 primary trial was not designed to investigate the influence of concomitant infection on vaccine-induced immune response and should be interpreted cautiously.

**Trial registration:**

WHO ICTRP, PACTR201411000934120.

## 1 Introduction

Malaria is a huge global public health problem with around 40% of the world’s population at risk of infection. In 2020, there were approximately 241 million clinical episodes of malaria and more than 627,000 deaths (1). Most of the mortality occurs among children under 5 years of age in sub-Saharan Africa where it is estimated that a child dies from malaria every minute. Malaria is caused by five species of parasites that infect humans, and all these species belong to the genus *Plasmodium*: *P. falciparum*, *P. vivax*, *P. ovale*, *P. malariae* and *P. knowlesi*. Malaria due to *P. falciparum* is the deadliest form and predominates in Africa.

An effective malaria vaccine is crucial in the face of continued high malaria transmission, increasing drug and insecticide resistance, and challenges in implementing different intervention strategies, e.g. bednets and indoor residual spraying (2–6). To date, only RTS,S, a pre-erythrocytic stage vaccine, has shown partial protection and is now recommended to be used for the prevention of *P. falciparum* malaria in children living in regions with moderate to high transmission, as defined by WHO (7, 8). The development of next-generation more effective malaria vaccines remains a high priority and is necessary for the global eradication of malaria.

There is a strong justification for blood-stage vaccines. Indeed, unlike vaccines against the pre-erythrocytic stages, vaccines against the blood-stage aim at reducing the parasite load by preventing invasion of red blood cells or limiting parasite replication/growth after invasion, thus protecting against clinical malaria. So far, reported vaccine candidates that target the blood-stage inhibit the growth of the parasites largely by allele-specific antibodies (9).

A recombinant form of SERA5 N-terminal domain (SE36) was selected for clinical development based on (a) epidemiological studies that show correlations of high antibody titers and malaria symptoms and severe disease (10–13); (b) *in vitro* studies that demonstrate parasite growth inhibition or antibody-dependent complement-mediated lysis of schizonts, or antibody-dependent monocyte-mediated parasite growth inhibition (14–17); and (c) non-human primate challenge studies that demonstrate protection against *P. falciparum* challenge infection (10, 17, 18). The serine repeat antigen 5 (SERA5) is a highly expressed, essential blood-stage protein (19) with limited genetic differentiation and polymorphism (20). SE36 was prepared under Good Manufacturing Practice (GMP) standards and formulated with aluminum hydroxide gel (AHG) to yield BK-SE36 (10).

Phase I safety and immunogenicity trials of BK-SE36 were conducted in healthy, malaria-naïve Japanese adults (10), in malaria-exposed Ugandan volunteers aged 6- to 32-year-old (21), and in healthy 12- to 60-month-old Burkinabe children (22). These trials have demonstrated the safety and immunogenicity of BK-SE36.

In the effort to optimize vaccine induced immune response and identify factors that can confound trial endpoints, or influence vaccination schedules, in this study we aimed at understanding whether malaria infection at the time of vaccination may explain some of the heterogeneity observed in initial trials. Thus, we examined the malaria blood smears collected before each vaccination during the primary trial to explore if *P. falciparum* infection had interfered with the immune responses to the BK-SE36 malaria vaccine candidate in line with previously reported malaria-induced immunosuppression (23, 24).

## 2 Methods

### 2.1 Immunization and sample processing

The full study methodology as well as the trial protocol were published elsewhere (22). In brief, a double-blind, randomized, controlled, single-center study was conducted in Banfora, a place with intense and markedly seasonal malaria transmission where *P. falciparum* is responsible for more than 90% of all clinical malaria cases (25, 26). Coinciding with the rainy months of May-November, 60% of clinical cases are reported during the months of June-September (26). The age-de-escalating, phase Ib clinical trial with a single-blind follow-up phase enrolled 108 healthy, malaria-exposed African children. Children in both Cohort 1 (aged 25–60 months, n = 54) and Cohort 2 (aged 12–24 months, n = 54) were randomized into 3 treatment arms in a 1:1:1 ratio receiving: (a) 3 full doses of BK-SE36 by the subcutaneous route (SC), (b) 3 full doses of BK-SE36 by the intramuscular route (IM), and (c) 2 doses of the licensed *Pneumococcal* polysaccharide conjugate decavalent Synflorix^®^ vaccine, alternate with 1 dose of physiological saline by the IM route (control arm). The inclusion of a third dose at Week 26 (W26; Day 182) was intended to increase the immune response and evaluate the effect of a booster dose. The first and second vaccine doses were given during the rainy season (peak of malaria transmission); the third dose during the dry (low transmission) season. In Cohort 1, the primary doses (28 days apart) were given in July-Aug 2015 and the booster dose (6 months from the first dose) given January 2016. For Cohort 2, primary vaccinations were given Oct-Nov 2015, and the booster on April 2016.

Anti-SE36 IgG antibody titers were measured by ELISA before vaccination (D0, D182), 4 weeks after each vaccination (D28, D56, D210), and at D365 and D477. ELISA was outsourced to a GLP certified testing facility (CMIC Pharma Science Co., Ltd., Japan) and used a standardized methodology. Results were expressed in titers calculated using an equilibrium line assay (10, 21).

Geometric mean titers (GMTs) were calculated at each time point. IgG1 and IgG3 subclasses were determined (27) for those with detectable anti-SE36 antibody titers 4 weeks after the second (W8/D56) and third (W30/D210) vaccinations.

Prior to each vaccination, capillary blood was also collected to prepare malaria smears. The presence of parasites was assessed by 100 x bright field microscopic examination, assuming 8,000 leukocytes/µl of blood. The count was made by species (*P. falciparum*, *P. malariae* or *P. ovale*), and counts for *P. falciparum* were made for both sexual and asexual parasites. The parasite presence and density were determined independently by two readers for the same slide; if readings were judged to be discordant, a third independent read was organized. The parasite density (parasites/μL) was calculated as the geometric mean of the two positive readings (two geometrically closest readings in the case of three positive reads). Microscopist competency was evaluated through two external quality control (EQC) programs. The first EQC was carried out by the College of American Pathology; proficiency testing with a set of 5 slides provided to each microscopist for reading thrice a year. Only a performance graded above 80% was considered satisfactory for the laboratory. The second EQC was performed by WHO (National Institute for Communicable Diseases) and involved the reading of a set of 20 slides every quarter by each microscopist. Only those with a score of at least 80%, graded as ‘competent’, were tasked in the reading of trial participants’ slides.

### 2.2 Statistical methods

Since the primary analysis showed no difference in terms of immune responses between the intramuscular and subcutaneous routes (22), the two arms were pooled for this study. The control arm is not of interest and was excluded from the current secondary analysis.

There were three categories depending on vaccination days and available blood smears/parasitemia data on those days. The first category (subgroup 1) includes subjects who received Dose 1 and 2 (termed “primary immunization”) and had a blood smear taken at these two occasions with results available. The subgroup considered subjects who received 2 doses since the interest is to see the effect of infection on the full vaccination status; however, the difference in GMTs between infected and non-infected subjects after one dose is provided in **Supplementary Table S1**. The second category (subgroup 2) is composed of subjects who received the booster (Dose 3) vaccination and had results of a blood smear taken before the booster dose. The third category (subgroup 3) includes subjects that received both primary and booster vaccinations and had results available for blood smear obtained at each of the three vaccination days.

Antibody titers and *P. falciparum* count are presented in terms of the geometric mean with 95% confidence interval. Because SE36 antibody titers are not normally distributed (with outliers as well as limits of detection (*e.g.*, undetectable titers were assigned a value of “8” for statistical analyses), we use the Wilcoxon rank sum test. All the comparisons are made between infected and uninfected groups. A *p*-value of 0.05 was the level of statistical significance.

Statistical analyses were performed using Stata ver. 14 (StataCorp, College Station, TX, USA, www.stata.com) and GraphPad (Prism ver. 9.4.0).

### 2.3 Ethical and regulatory approval

The trial was conducted according to the principles of the Declaration of Helsinki and the International Conference on Harmonization (ICH) Good Clinical Practice (GCP) guidelines. The clinical trial protocol and associated documents were reviewed and approved in Burkina Faso by CNRFP institutional bioethics committee (Ref: n°2014/071/MS/SG/CNRFP/CIB, N°2016/000008/MS/SG/CNRFP/CIB), the Ministry of Health Ethical Committee for Biomedical Research (Ref: 2014-12-144) and the National Regulatory Authority (Approval for the clinical trial: (N°2015_658/MS/CAB). The trial package was also approved by the Scientific Committee/Institutional Review Committee of the Research Institute for Microbial Diseases (Ref: 26-6), Osaka University (Ref: 574) in Japan; and the London School of Hygiene and Tropical Medicine Research Ethics Committee (Ref: 9175) in United Kingdom.

## 3 Results

### 3.1 Baseline characteristics

The vaccination of Cohort 1 started on July 4, 2015 (Dose 1), and the last vaccination (Dose 3) was completed by January 11, 2016. Vaccination of Cohort 2 began on October 12, 2015 (Dose 1) and was completed by April 18, 2016 (Dose 3). In total 72 participants received 2 primary doses of BK-SE36 (22), however 1 subject in Cohort 1 had a missing blood smear during Dose 2 vaccination day and thus only 71 subjects were included in subgroup 1 analyses. For subgroup 2, all subjects who have received Dose 3 and had a blood smear before vaccination were included in the analyses (n=68). For subgroup 3, all subjects with primary and booster vaccination and a blood smear at these vaccination days were included (n=68).

The median age was 25.4 (IQR 28.2) months, and 38.9% of subjects were male.

### 3.2 Carriage of *Plasmodium falciparum* infection during immunization

**Figure 1:**
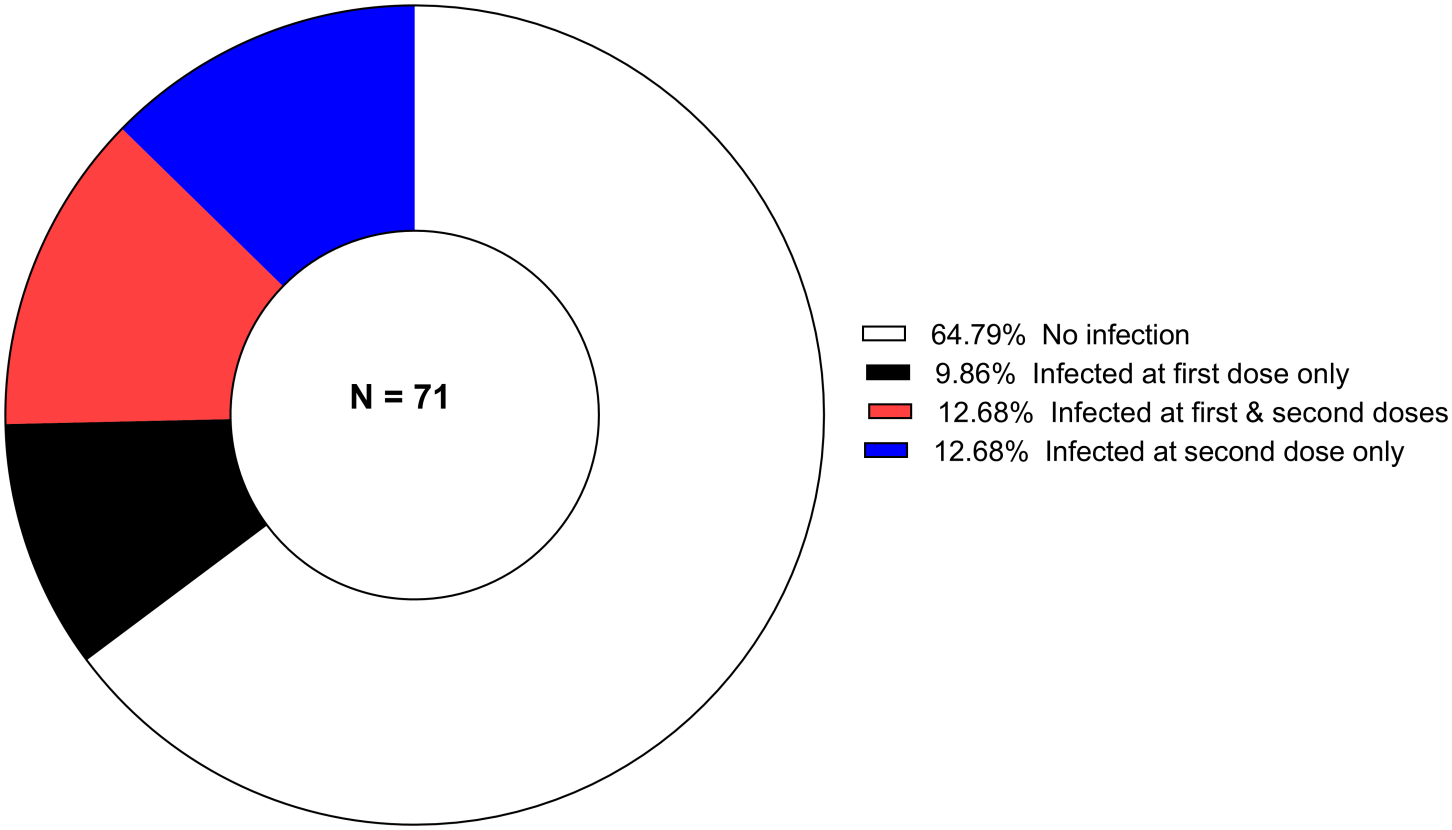
The proportion of *P. falciparum-*infected children during primary vaccination (Dose 1 and Dose 2) days.

The proportion of individuals carrying *P. falciparum* infection at the time of primary immunizations is shown in **Figure 1**. Infection is defined as any asexual parasitemia > 0 by microscopy. In this study, the vaccine treatment arms (SC and IM) were pooled for analysis due to the small sample size, the randomized nature of the trial, and the results indicating no difference in the vaccine induced immune response between the two routes. Therefore, it is unlikely that there are differences in malaria infection during vaccinations days and vaccine induced antibody response in the SC and IM arms. During vaccinations days there were no recorded *P. malariae*, *P. vivax* and *P. ovale* infections.

For subgroup 1, 64.8% (46/71) participants were free of infection at both Dose 1 and 2. At either dose, 35.2% (25/71) were harboring malaria parasites. The mean age was 39.6 ( ± 14.9) and 27.9 ( ± 14.6) months in infected and uninfected children, respectively. The difference in the proportion of children infected during vaccination days was statistically significant (*p* = 0.002).

In subgroup 2, no *P. falciparum* infection was found for 83.8% (57/68) of the vaccinees at the time of the booster vaccination. The mean age was 45.2 ( ± 14.6) and 29.5 ( ± 14.9) months in infected and uninfected children, respectively. The difference was statistically significant (*p* = 0.001).

In total, 58.8% (40/68) receive their primary and booster doses in the absence of any *P. falciparum* infection (subgroup 3). The mean age was 39.7 ( ± 15.8) and 26.7 ( ± 13.8) months in infected and uninfected children, respectively. Again, the difference was statistically significant (*p* < 0.001).

The geometric mean of *P. falciparum* parasites density was 1077.6 trophozoites/µL (95% CI, 451.5-2571.9), 1811.4 trophozoites/µL (95% CI, 4716.9-4576.8) and 2359.9 trophozoites/µL (95% CI, 649.3-8577.6) respectively at Dose 1, 2 and 3. Individual *P. falciparum* count is provided in **Supplementary Figure S1**.

### 3.3 Immune responses and *Plasmodium falciparum* carriage status

**Figure 2:**
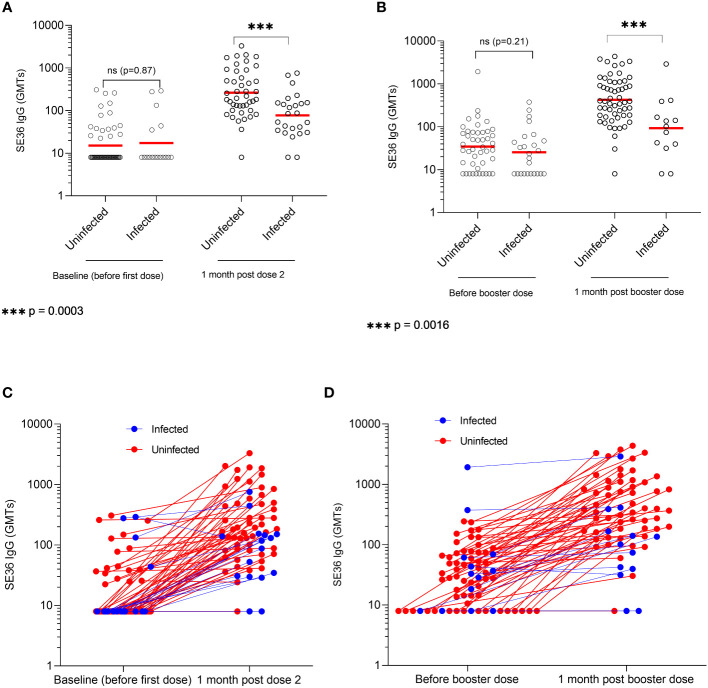
SE36 antibody response by infection status. Panels **(A, B)** show the serum anti-SE36 antibody titers (expressed as geometric means, GMT) at primary and booster vaccination, respectively. Panels **(C, D)** show the individual change in antibody titers one month after Dose 2 and a booster dose (Dose 3), respectively. Solid red line represents the geometric mean.

Immune responses to BK-SE36 vaccination in infected and uninfected individuals are shown in **Figure 2**. At baseline (before Dose 1, **Figure 2A**), the GMT of SE36 antibody titers was 15.1 (95% CI, 11.3-20.2) in uninfected individuals compared to 17.3 (95% CI, 8.7-34.4) in infected participants (*p* = 0.877). One-month post-primary vaccinations (after Dose 2), the geometric mean of SE36 antibodies was 263.2 (95% CI, 178.9-387.1) in uninfected individuals compared to 77.1 (95% CI, 47.3-125.7) in infected participants. The difference was statistically significant (*p* < 0.001). In terms of fold change for each subject, there was a 2.2 (95% CI, 0.1.3-3.5) and 1.6 (95% CI, 1.0-2.6) fold change respectively in uninfected and infected participants from baseline to one-month post Dose 2. The difference, however, was not statistically significant (*p* = 0.361, **Figure 2C**).

Before Dose 3 or booster dose (22 weeks post Dose 2), the GMT was 34.6 (95% CI, 24.3-49.3) in uninfected individuals compared to 25.4 (95% CI, 15.4-41.9) in infected participants. The difference was not statistically significant (*p* = 0.212) (**Figure 2B**). One month post Dose 3, participants uninfected at the time of booster vaccination had significantly higher GMTs compared to those who were infected on vaccination day (424.1 (95% CI, 301.9-595.8) vs. 92.8 (95% CI, 34.9-246.6); *p* = 0.002). There was a 14.3 (95% CI, 9.7-21.1) and 2.4 (95% CI, 1.3-4.4) fold change respectively in uninfected and infected participants between one-month post Dose 2 and Dose 3. The difference was statistically significant (*p* < 0.001, **Figure 2D**).

Study participants who did not have any microscopically detectable *P. falciparum* infection during primary and booster vaccinations had the highest GMT at one-month post Dose 3 (602.2; 95% CI, 421.5-860.3) compared to those who were found with *P. falciparum* infection at some point in time during vaccination days (139.2; 95% CI, 80.7-240.3). The difference between uninfected and infected subjects was statistically significant (*p* < 0.001). This difference was maintained towards the study end with GMTs of 101.0 (95% CI, 62.3-163.8) vs 27.21 (95% CI, 15.5-44.5) (*p* = 0.006) at visit 32 (D365); and 96.7 (95% CI, 59.4-157.3) vs. 25.6 (95% CI, 15.5-42.4), (*p* = 0.001) at visit 36 (D477) (uninfected vs infected subjects). A subgroup analysis by cohort is provided in **Supplementary Table S2**.

Anti-SE36 IgG antibody subclasses levels in uninfected and infected children are presented in **Supplementary Figure S2**. The IgG1 concentrations measured 4 weeks after Doses 2 and 3 were significantly higher in uninfected children as compared to those who had an infection during vaccination days. For IgG3, a statistically significant difference in titers between both groups was only observed post-Dose 2.

## 4 Discussion

In this study, we report the immune responses post-vaccination with BK-SE36 blood-stage malaria vaccine candidate in 12-60 months old children in Burkina Faso. The study findings suggest that concurrent blood-stage infection during immunization/vaccination days has reduced the magnitude of the immune responses to the vaccine. This low response to vaccination in infected individuals has also been reported for pre-erythrocyte vaccine candidates including PfSPZ (28) and ME-TRAP (29). In another phase 1 trial with a blood-stage vaccine candidate, Combination B (comprised of three asexual blood-stage antigens: merozoite surface protein 1, merozoite surface protein 2 and the ring-infected erythrocyte surface antigen), the effect of parasite clearance with sulphadoxine-pyrimethamine (SP) prior to vaccination in 5-9 years-old Papua New Guinean children was assessed (30). Antibody titers to the component antigens were not significantly modified/enhanced by SP treatment 1 week prior to vaccination although cellular response (IFN-γ) to merozoite surface protein 1 was substantially lower in the vaccinated subjects who had received SP. Additional evidence will be needed to better understand the differences observed between the BK-SE36 and Combination B vaccines. It shall also be noted that the BK-SE36 primary trial was not designed to investigate the influence of concomitant infection on vaccine-induced immune response and should be interpreted cautiously.

The malaria parasite is known to induce immunosuppression in the host during asexual/blood-stage development and this impairs the generation as well as the function of humoral and cell-mediated immunity (31). Molecular profiling has shown that asymptomatic *P. falciparum* malaria is characterized by an important immunoregulatory blood transcriptional signature with the upregulation of various pathways involved in the inhibition of CD4^+^T-cell function (32). As such, asymptomatic malaria might not support the induction of immune processes to control parasitemia or respond efficiently to malaria vaccines.

SE36 is known to tightly bind to host prote*in vitro*nectin that further binds to different host proteins in an act of molecular camouflage (33). Repeated infections and the presence of these vitronectin-bound SE36 complexes could inevitably result in immune tolerance against SE36 molecule. The reported immune tolerance is mainly due to a host response to repeated infections and takes some time to acquire (33, 34). The immunosuppression described here would rather be caused by an impaired vaccine-induced immune response consequent to a concomitant infection. It can be argued that, in the analysis, infection was defined as any asexual parasitaemia > 0 by microscopy and this definition could include new infections, those with rising parasitaemia or long-standing infections imperfectly controlled by host immune response. The primary trial was designed to have two age cohorts (22). Cohort 1 with children aged 25-60 months and a younger Cohort 2 with children aged 12-24 months. The children of Cohort 1 have presumably a longer history of exposure to repeated malaria infections, and some may represent a subgroup of children with lower SE36 responsiveness. The immune tolerance of Cohort 1 may explain why the younger children of Cohort 2 had 2-fold and 4-fold higher antibody titers than Cohort 1 after Dose 2 and Dose 3 of BK-SE36, respectively (22). In the BK-SE36 arms before vaccination, there were more children in Cohort 2 that presented high antibody titres (n=17) than in Cohort 1 (n=5). This finding may be due to chance or presence of maternal antibodies in younger cohorts [as explained in (22)]. Another observation was that more children in Cohort 1 were infected at the time of Dose 1 and 2 (52.8%) *vs*. 19.4% in Cohort 2, *p* = 0.003. Most likely, the observed lower immune response in Cohort 1 may be due to immune suppression. The response during or shortly after infection with *Plasmodium* spp. was reviewed by Calle et al. (31). Immunosuppression is likely a complex combination/interplay of innate and adaptive immune factors that may involve qualitative changes in B- and T-cell responses (35), *e.g.* expansion of atypical memory B cells (36), reduction of T-cells (37–39), activation of NK cells for early IFN-γ response (40).

Notably, not only the absolute quantity of antibody but the quality (affinity and subclass) and longevity of antibodies are also important (41, 42). Antibody levels dropped to near pre-vaccination titers 6 months after the first dose in both uninfected and infected participants (GMT 25 and 34). After Dose 3, antibody levels peaked 4-fold higher than after Dose 2, suggesting that a third dose is necessary in endemic areas to obtain the best possible immune response (a suggestion that was inferred also from the Ugandan study (21)).

Of note, a multivariate analysis showed, as expected, a negative correlation between parasite density and anti-SE36 antibody GMT. Adjusted for baseline antibody titer, age, and the interaction between age and baseline antibody response there was a 1.93%, 133.9% and 11.3% decrease in antibody GMT for each 1000 parasites/μl increase in *P. falciparum* density, respectively for subgroup 1, 2 and 3. Whether clearing the infections before immunization could mitigate this immunosuppression also needs further studies.

Currently, the status of *P. falciparum* infection was only defined based on microscopy. Owing to the detection limit of optical microscopy (~ 100 parasites/µL), we could not rule out the possibility that submicroscopic infections were missed (43), as this is a common feature in malaria-endemic settings (44–46). Moreover, because of the dynamic parasite life cycle, the time of blood sampling may also miss low-density infections even if current routine molecular diagnostic tests are used (46). While misclassifications of the subgroup may bias estimates, the observation that concomitant parasitemia (not just a history of infection) is not innocuous has implications for the scheduling and administration of the malaria blood-stage vaccine BK-SE36.

## 5 Conclusions

These findings confirm the immunosuppressive role of *P. falciparum* and the low response caused by parasite infection when the BK-SE36 malaria vaccine candidate is administered. The study also supports the need to further explore whether clearing the existing infections before vaccination could improve immune responses and potential vaccine efficacy.

## Data availability statement

The original contributions presented in the study are included in the article/**Supplementary Material**. Further inquiries can be directed to the corresponding authors.

## Ethics statement

The studies involving human participants were reviewed and approved by Burkina Faso : CNRFP institutional bioethics committee (Ref: n°2014/071/MS/SG/CNRFP/CIB, No2016/000008/MS/SG/CNRFP/CIB), the Ministry of Health Ethical Committee for Biomedical Research (Ref: 2014-12-144) and the National Regulatory Authority (Approval for the clinical trial (No2015_658/MS/CAB); Japan: the Scientific Committee/Institutional Review Committee of the Research Institute for Microbial Diseases (Ref: 26-6), Osaka University (Ref: 574); and United Kingdom: London School of Hygiene and Tropical Medicine Research Ethics Committee (Ref: 9175). Written informed consent to participate in this study was provided by the participants’ legal guardian/next of kin.

## Author contributions

SS, EB, AT, IN, SH, FD, NP, and TH contributed to the conception and design of the study. AT, EB, IN, AO, SS were responsible for study implementation at the study site. AT performed the statistical analysis. NA and NP for materials/reagents/assay development and analysis tools. AT and NP wrote the first draft of the manuscript. All authors contributed to the article and approved the submitted version.
