# Supplementary material for: Plasmodium falciparum infection coinciding with the malaria vaccine candidate BK-SE36 administration interferes with the immune responses in Burkinabe children
Source: Front Immunol. 2023 Mar 10;14:1119820. doi: 10.3389/fimmu.2023.1119820 (PMC10040972; doi:10.3389/fimmu.2023.1119820)

Supplementary Material

1 Supplementary Table

Supplementary Table S1: SE36 antibody responses according to study cohort and infection status at one-month post first dose

Timepoints	Cohort 1 (25–60 months)	Cohort 2 (12–24 months)	***P***
	**GMT (95% CI)**	**GMT (95% CI)**	
**Uninfected**	23.3 (13.6-39.7)	67.4 (46.4-98.0)	0.0033
**Infected**	23.1 (10.7-49.9)	31.5 (0.02-46846.5) **	0.529

** only two individuals were included in this analysis

Supplementary Table S2: SE36 antibody responses according to study cohort and infection status

Subgroups	Timepoints	Cohort 1 (25–60 months)	Cohort 2 (12–24 months)	*P*
		**GMT (95% CI)**	**GMT (95% CI)**	
**Subgroup 1**	***Visit 2 (before first dose)***			
	Uninfected	10.3 (7.0-15.2)	20.1 (12.4-32.6)	0.03
	Infected	13.5 (7.4-24.7)	22.1 (11.4-42.9)	0.03
	Visit 18 (1-month post Dose 2)			
	Uninfected	151.8 (81.7-281.9)	372.2 (231.2-599.1)	0.03
	Infected	73.7 (39.7-136.7)	88.9 (35.1-225.5)	0.7
**Subgroup 2**	***Visit 19 (before booster dose)***			
	Uninfected	45.9 (28.8-73.2)	21.5 (15.3-30)	0.01
	Infected	25.2 (11.4-55.8)	355.9 (2.54e-07-5.26e+11) **	0.04
	Visit 27 (1-month post booster dose)			
	Uninfected	257.2 (171.5-385.9)	617.2 (378.7-10005.8)	0.003
	Infected	59.6 (25.9-136.8)	1062 (0.0031291-3.61e+08) **	0.04
**Subgroup 3**	***Visit 27 (1-month post booster dose)***			
	Uninfected	316.3 (184.8-541.3)	863.7 (563.5-1323.9)	0.006
	Infected	104.2 (59.1-183.7)	273.7 (70.3-1066.2)	0.09
	Visit 32			
	Uninfected	50.2 (11.5-116.9	149.5 (84.4-264.7)	0.01
	Infected	24.4 (13.9-42.6)	34.7 (10.3-116.9)	0.62
	Visit 36			
	Uninfected	130.7 (522-327.5)	81.6 (44.7-148.9)	0.25
	Infected	23.4 (14.4-38.1)	31.6 (7.3-137.0)	0.98

** only two individuals were included in this subgroup

2 Supplementary Figure

Supplementary Figure 1: *P. falciparum* parasites density at the immunizations timepoints. (Cohort 1: 25-60 months-old; Cohort 2: 12-24 months-old)

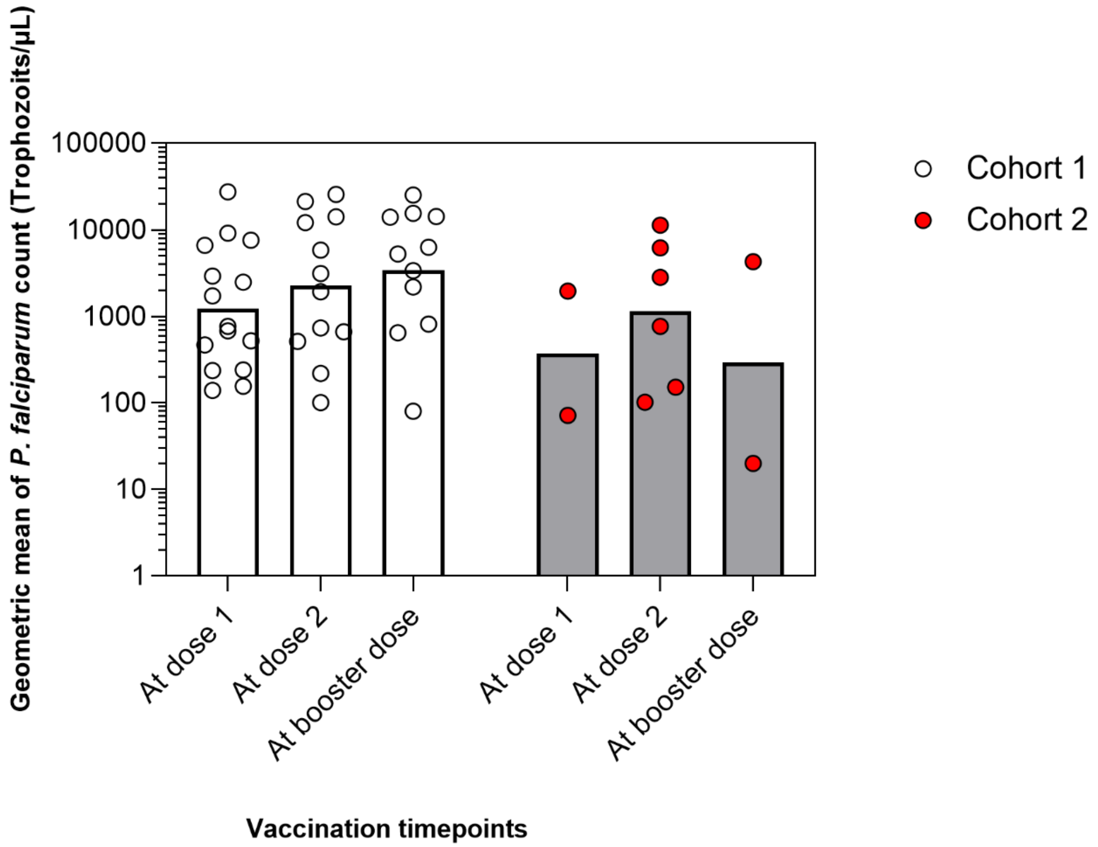

Supplementary Figure 2: SE36 antibody response by infection status. Panel **A** shows the serum anti-SE36 antibody titers (expressed as geometric means, GMT) at one month post primary vaccination (Dose 2) for IgG1 and IgG3 in subgroup 1 children. Panel **B** shows the GMT at one-month post booster vaccination for IgG1 and IgG3 in subgroup 2 participants. Solid red line represents the geometric mean.

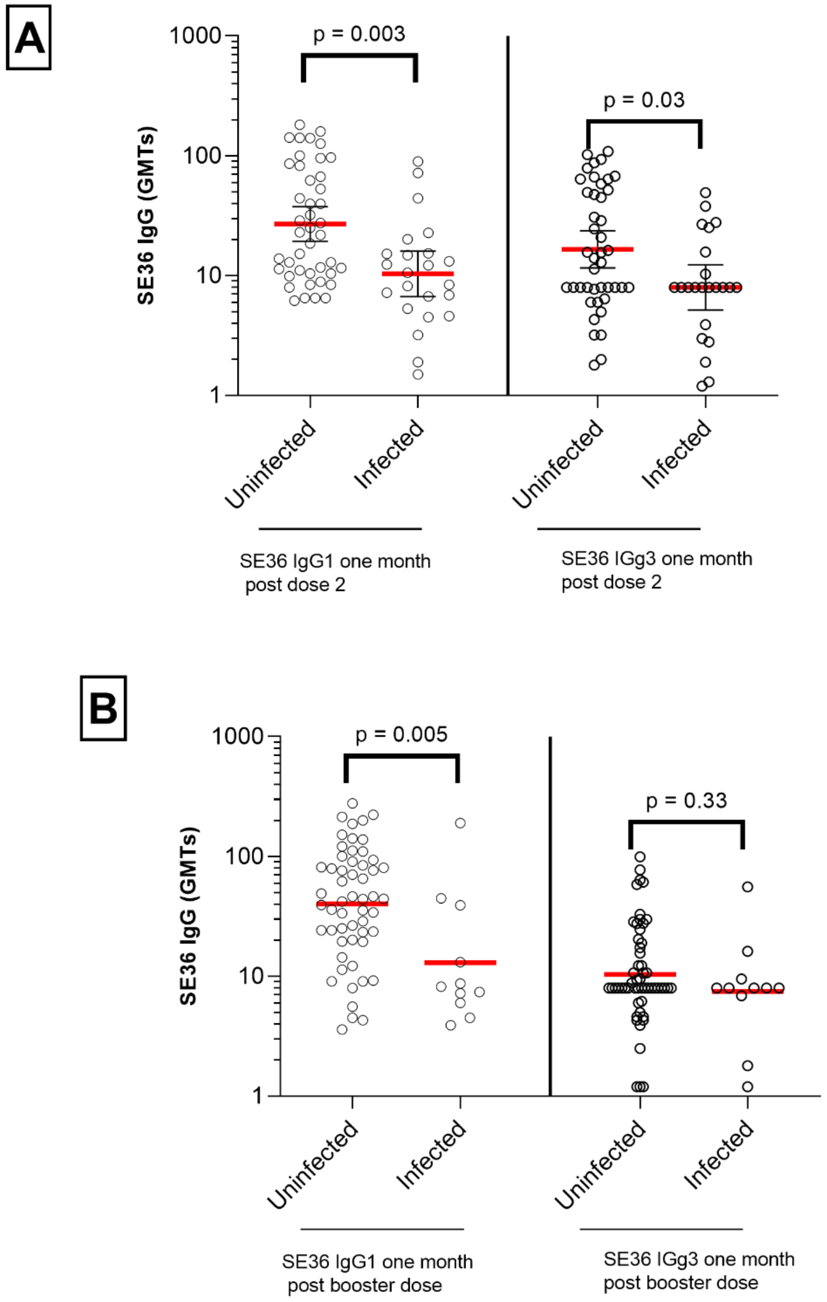

Supplement: Supplementary file 1 [file DataSheet_1.pdf]
